# HAV infection in Brazilian men who have sex with men: The importance of surveillance to avoid outbreaks

**DOI:** 10.1371/journal.pone.0256818

**Published:** 2021-09-16

**Authors:** Lisie Souza Castro, Grazielli Rocha de Rezende, Fernanda Rodas Pires Fernandes, Larissa Melo Bandeira, Gabriela Alves Cesar, Barbara Vieira do Lago, Michele Soares Gomes Gouvêa, Ana R. C. Motta-Castro

**Affiliations:** 1 Federal University of Mato Grosso do Sul, Campo Grande, Brazil; 2 Federal University of Mato Grosso, Rondonópolis, Brazil; 3 Ministry of Agriculture, Livestock and Food Supply/National Agricultural Laboratory, MAPA/LANAGRO, Minas Gerais, Brazil; 4 Laboratory of Viral Hepatitis, Oswaldo Cruz Foundation, FIOCRUZ, Rio de Janeiro, Brazil; 5 Institute of Tropical Medicine, University of São Paulo, São Paulo, Brazil; 6 Oswaldo Cruz Foundation, FIOCRUZ, Rio de Janeiro, Mato Grosso do Sul, Brazil; FIOCRUZ, BRAZIL

## Abstract

**Background:**

Hepatitis A is a fecal-oral infection caused by hepatitis A virus (HAV). Men who have sex with men (MSM) and transgender women (TW) have been reported as target groups for HAV infection. This study aimed to determine the seroprevalence, risk factors, and circulating strains associated with HAV infection among MSM and TW in Central Brazil.

**Methods:**

A cross-sectional study was conducted from November 2011 to September 2013. Serum samples were collected from 425 individuals for anti-HAV antibody testing and HAV molecular characterization. Of them, 149 (35.1%) participants were self-identified as transgender women. Statistical analysis was performed to evaluate the risk factors of HAV seropositivity.

**Results:**

The seroprevalence of HAV exposure was 69.7% (95% Confidence Interval: 65.3–74.0%). Serological evidence of HAV was significantly higher in participants who self-identified as transgender women (83.2%) than MSM (62.3%). Increasing age, non-white race, and lower monthly household income were independently associated with HAV exposure among MSM. Only lower monthly household income was independently associated with HAV exposure among TW. One anti-HAV IgM positive sample, from a transgender woman (0.2%), was detected and classified as subgenotype IA.

**Conclusions:**

High HAV prevalence was observed, markedly among TW. Considering the risky sexual behaviors this population is exposed to, HAV vaccination and prevention programs targeting this population should be considered to prevent outbreaks and the burden of the disease.

## Introduction

Hepatitis A virus (HAV) infection represents a significant public health problem worldwide. Prevalence rates are closely related to socioeconomic factors that impact the quality of access to potable water and sanitation [1]. At the early stages of life, HAV infection is usually asymptomatic; by contrast, about 70% of adults develop symptomatic hepatitis. Transmission occurs through the fecal-oral route via ingestion of contaminated food or water and direct person-to-person contact, including sexual (oro-anal, oro-genital, and digital-anal) contact [1, 2].

HAV seroprevalence varies according to geographical and socioeconomic regions. Until the start of infant mass vaccination, Brazil had an intermediate endemicity [3]. However, studies with mathematical models pointed to a decrease from an intermediate to a low incidence rate, with the shift of the age of infection to adulthood [1]. This epidemiological scenario may cause a significant impact on the burden of disease, especially in high-risk groups [1, 4].

Specific groups are at high risk to HAV infection, such as travelers to endemic areas, injecting-drug users, men who have sex with men (MSM) [4]. The main risk factor among MSM is related to sexual practices and risk behaviors, classifying HAV as a sexually transmitted infection (STI) [2, 4, 5]. Nowadays, several HAV outbreaks have been reported in the MSM population across Europe and Americas (Chile and the USA) [4]. In Brazil, a men’s HAV outbreak occurred in São Paulo, in which 44% of infected individuals were MSM [6].

In 2016, the Brazilian Ministry of Health conducted the National Biological and Behavioral Surveillance Survey (BBSS) among MSM in which investigated HIV, HBV, HCV, and syphilis infections but not HAV infection [7]. Since the infection can be severe in adults [1], the epidemiological study of HAV infection among MSM may contribute to the implementation of preventive measures in this target group. The vaccine against HAV was included in the Brazilian public immunization program in August 2014. However, only children between one and five years of age are currently targeted for vaccination [6]. Young individuals and adults remain susceptible to HAV.

Therefore, considering the lack of studies on HAV infection in MSM from Central Brazil, this study aimed to estimate the prevalence and the risk factors to HAV infection, as well as to identify circulating viral strains among MSM, a high-risk population to acquiring this infection.

## Materials and methods

This cross-sectional study was conducted in Campo Grande, Mato Grosso do Sul, Central Brazil. This study was approved by the Ethics Committee on Human Research of the Federal University of Mato Grosso do Sul, under protocol number 1.250.214 –CAAE: 49368115.9.0000.0021. The recruitment of MSM and TW convenience sampling was carried out between November 2011 to September 2013, at public (e.g., gay pride parades, square, parks, streets, etc.) and private places (e.g., bars, massage parlors, saunas, nightclubs, brothels, etc.) of the city. MSM and TW were previously contacted through the Mato Grosso do Sul State Association of Travestites and Transsexuals and Reference Center for Human Rights in the Prevention and Combat of Homophobia. Informed consent about the study was obtained and signed from all participants. As described by Fernandes et al. (2014), the study participants were divided into two groups, MSM and TW, according to the self-designation of the individuals during the interview. TW was defined as a group of individuals who were born male but who are identified as a different gender. In Brazil, they include crossdressers or transvestites (those who desire to wear clothing associated with another sex) and male-to-female transexuals (those who desire or have undergone sexual reassignment surgery or altered their bodies with hormone therapy and silicone injections).

MSM and TW who were 18 years or more and reported sexual intercourse with another man within 12 months before the day of the interview were eligible to participate in the study. Face-to-face interviews with a structured standardized questionnaire were conducted to collect socio-demographic characteristics (age, marital status, educational level, household income) and risk behaviors for HAV transmission, including drug use, alcohol consumption, sexual practices, and syphilis status. Right after the interview, blood samples were collected for IST screening from all participants and stored at -20°C. The sample size was calculated by hand using the following formula cited in the review Power and Sample Size determination by Lisa Sullivan. (https://sphweb.bumc.bu.edu/otlt/MPH-Modules/BS/BS704_Power/BS704_Power_print.html), based on the prevalence of 42.3% for total anti-HAV among MSM [8], significance level of 95% (α <0.05), and 5% of alpha-type error. Thus, the study should include at least 377 individuals, and the study population was 425 individuals.

Serum samples were tested for IgM and total anti-HAV antibodies by enzyme immunosorbent assay (ELISA-DiaSorin, Italy). Syphilis infection was screened by ELISA (ICE Syphilis, DiaSorin, UK). Anti-HAV IgM positive sample was submitted to HAV-RNA detection and sequencing with primers for VP1/P2A region as described previously [9]. Nucleotide sequence analyses were performed using a dataset composed of 87 VP1-2A region sequences with 216 nucleotides from genotype IA, representing the main HAV worldwide outbreaks. Phylogenetic comparisons were accessed using the Maximum Likelihood method, bootstrap resampling test with 1000 replicates, under General Time Reversible model with gamma distributed rate heterogeneity (GTR+G), implemented in Mega software v.7.0.

Statistical analyses were performed using STATA, version 13. Prevalence was calculated with 95% confidence interval (CI). Categorical parameters were analyzed for comparison by Chi-square and Fisher’s exact tests. Multiple logistic regression model was used additionally in parameters that were significantly associated with anti-HAV positivity (p<0.20). All reported values are two-tailed. The selection of variables for the final model was performed stepwise, according to the number of events per variable (EPV). Hosmer-Lemeshow test was used to assess goodness-of-fit, choosing the best regression equation [10]. A value of p < 0.05 was considered statistically significant.

## Results

### Study population characteristics

During the study period, 535 individuals were invited to participate, 430 (80.4%) agreed to answer the questionnaire. Among these, 425 (98.8%) provided blood samples to perform the analysis and were included in the study. The median age of MSM (n = 276) was 23, ranging from 18 to 61 years old. Among TW (n = 149), the median age was 26, ranging from 18 to 70 years old. Both groups (91.3% of MSM and 79.8% of TW) were predominantly unmarried (single, divorced, or widowed) and 12.6% of all participants reported as being married to MSM or women. Less than 9 years of schooling was reported by 39.1% of MSM and 51% of TW.

Lower monthly household income (≤ 264.00 USD) was reported by 14.6% and 39.4% of MSM and TW, respectively. Frequent consumption of alcohol was reported by TW (65.1%) and MSM (67.6%). Recreational drug use differed substantially by group (p<0.001). Use of non-injecting illicit drugs was reported by 48.5% of all participants (MSM and TW), with marijuana being the most commonly consumed drug (77.1%). Three (1.1%) MSM and TW (1.3%) reported the use of injecting illicit drugs.

Exchange of sex for money or goods was more common among transgender women than MSM (75.8% vs. 15.9%; p<0.001). Almost fifty-three percent of MSM had sexual debut before 15 years old and the mean age was 15.7 years (standard deviation [SD] = ±2.98). Among TW, 69.8% reported sexual debut before 15 years old and the mean age of sex work initiation was 16.4 years (standard deviation [SD] = ±3.43). Regarding the number of sexual partners in the last seven days before data collection, 23.8% of MSM reported two or less than 10 partners. Among TW, 46.3% of them reported more than 10 partners in the last seven days before data collection. Sex with a female partner in the past 12 months was reported by 37% of MSM and the absence of condom use at the last sexual intercourse was reported by 41.9% of all participants.

A history of ever having engaged in a variety of sexual practices was reported by 35.8% of all participants. The frequency of diverse sexual practices was as follows: rimming (53.6%), sadism and/or masochism (35.9%), ‘golden showers/water sports’ (28.1%), group sex (10.5%), and others (20.3%) (fisting, shared sex toys and others). Experiencing sexual coercion was reported by 24.5% of TW and by 13.6% of MSM.

### Anti-HAV prevalence and risk factors

Serological evidence of HAV infection (total anti-HAV antibodies positivity) was found in 296 individuals (69.7% 95% CI: 65.3–74.0) of the studied population. One sample tested positive for IgM anti-HAV. Prevalence of HAV infection among participants self-identified as transgender women (124/149, 83.2%) were significantly higher than MSM (172/276, 62.3%) (p<0.001). Tables 1 and 2 show sociodemographic characteristics and risk behaviors associated with anti-HAV positivity. All variables from the univariate analysis (p<0.20) were included in multiple logistic regression analysis. Increasing age [odds ratio (OR) 21.55; confidence interval (CI) 6.44–72.02], non-white race (OR 1.56; 95% CI 1.15–2.13) and lower monthly household income (OR 3.01; 95% CI 1.05–8.63) were independently associated with HAV exposure among MSM. Only lower monthly household income (OR 6.36; 95% CI 1.21–33.17) was independently associated with HAV exposure among TW.

**Table 1:** Analysis of socio-demographic and behavioral characteristics among MSM associated with positive anti-HAV antibodies (n = 276), Central Brazil.

Characteristics	Anti-HAV Positive*/Total (%)	Anti- HAV Negative*/Total (%)	Univariate analysis	Multiple logistic regression analysis
			*P* Value; OR (95% CI)	*P* Value; OR (95% CI)^b^
**Age (years)**				
<20	28/70 (40.0)	42/70 (40.0)	1.00	
20–24	56/99 (56.6)	43/99 (56.6)	0.035; 1.95 (1.05–3.63)	0.019; 2.46 (1.16–5.21)
25–29	36/50 (72.0)	14/50 (28.0)	0.001; 3.85 (1.76–8.42)	<0.001; 6.12 (2.37–15.80)
≥30	52/57 (91.2)	05/57 (8.8)	<0.001; 15.6 (5.54–43.90)	<0.001; 21.55 (6.44–72.02)
**Education (years)**				
>12	21/29 (72.4)	8/29 (27.6)	1.00	
10–12	92/139 (66.2)	47/139 (33.8)	0.517; 0.74 (0.30–1.81)	0.464; 1.60 (0.45–5.64)
≤9	59/108 (54.6)	49/108 (45.4)	0.089; 0.45 (0.18–1.12)	0.717; 0.78 (0.21–2.96)
**Marital status**				
Living with a partner	18/24 (75.0)	06/24 (25.0)	1.00	
Single	154/252 (61.1)	98/252 (38.9)	0.180; 0.52 (0.20–1.36)	0.797; 1.17 (0.33–4.15)
**Monthly household income (national minimum wage)** ^**#**^				
> 5	28/65 (43.1)	37/65 (56.9)	1.00	1.00
2–5	103/158 (65.2)	55/158 (34.8)	0.003; 2.47 (1.37–4.46)	0.333; 1.41 (0.69–2.88)
≤ 1	29/38 (76.3)	29/38 (23.7)	0.002; 4.25 (1.74–10.41)	0.039; 3.01 (1.05–8.63)
No Information (15)				
**Sex exchange for money and goods (ever)**				
No	135/232 (58.2)	97/232 (41.8)	1.00	1.00
Yes	37/44 (84.10)	07/44 (15.9)	0.002; 3.79 (1.62–8.87)	0.337; 1.87 (0.51–6.80)
**Number of sexual partners in the last week**				
≤ 1	122/210 (58.1)	88/210 (41.9)	1.00	
2–10	42/56 (75.0)	14/56 (25.0)	0.023; 2.16 (1.11–4.20)	0.876; 1.07 (0.45–2.54)
> 10	8/10 (80.0)	02/10 (20.0)	0.187; 2.88 (0.59–13.91)	0.877; 0.815 (0.06–10.84)
**Sexual practices** ^**a**^				
No	106/186 (57.0)	80/186 (43.0)	1.00	
Yes	50/71 (70.4)	21/71 (29.6)	0.050; 1.79 (0.99–3.23)	0.198; 1.60 (0.78–3.28)
No Information (19)				
**Experienced sexual coercion (ever)**				
No	128/223 (57.4)	95/223 (42.6)	1.00	
Yes	29/35 (82.8)	06/35 (17.2)	0.006; 3.58 (1.43–8.98)	0.051; 2.84 (0.99–7.96)
No Information (18)				
**Race/ethnicity**				
White	60/117 (51.3)	57/117 (48.7)	1.00	1.00
Non-White	112/159 (70.4)	47/159 (29.6)	0.001; 1.50 (1.17–1.92)	0.004; 1.56 (1.15–2.13)
**Total anti-HBc status**				
Negative	149/247 (60.3)	98/247 (39.7)	1.00	
Positive	23/29 (79.3)	06/29 (20.7)	0.052; 2.52 (0.99–6.41)	0.627; 1.33 (0.45–5.64)

OR: Odds Ratio; 95% CI: 95% Confidence Interval

^#^National minimum wage: during the study period, one minimum wage represented approximately R$ 998.00 BRL (U$ 264.00 USD).

* Positivity for total anti-HAV antibodies

^**a**^ Ever had engagement in sexual practices as rimming, group sex, shared sex toys and others

^**b**^ Adjusted for age, education and monthly household income.

**Table 2:** Analysis of socio-demographic and behavioral characteristics among TW associated with positive anti-HAV antibodies (n = 149), Central Brazil.

Characteristics	Anti- HAV Positive*/Total	Anti-HAV Negative*/Total	Univariate analysis	Multiple logistic regression analysis
			*P* Value; OR (95% CI)	*P* Value; OR (95% CI)^a^
**Age (years)**				
<20	19/24 (79.2)	05/24 (20.8)	1.00	1.00
20–24	34/45 (75.6)	11/45 (24.4)	0.735; 0.81 (0.25–2.69)	0.332; 2.12 (0.46–9.77)
25–29	24/29 (82,7)	05/29 (17.3)	0.740; 1.26 (0.31–5.01)	0.260; 2.74 (0.46–11.80)
≥30	47/51 (92.2)	04/51 (7.8)	0.119; 3.09 (0.74–12.77)	0.188; 3.34 (0.52–17.75)
**Monthly household income (national minimum wage)**				
> 5	8/13 (61.5)	05/13 (38.5)	1.00	1.00
2–5	59/70 (84.3)	11/70 (15.7)	0.066; 3.35 (0.02–2)	0.129; 3.25 (0.72–14.86)
≤ 1	49/54 (90.7)	05/54 (9.3)	0.014; 6.12 (1.44–26.04)	0.043; 6.14 (1.06–31.21)
No Information (12)				
**Race/ethnicity**				
White	28/39 (71.8)	11/39 (28.2)	1.00	1.00
Non-White	96/110 (87.3)	14/110 (12.7)	0.030; 1.64 (1.04–2.56)	0.528; 1.21 (0.67–2.18)
**Total anti-HBc status**				
Negative	87/110 (79.1)	23/110 (20.9)	1.00	
Positive	37/39 (94.9)	02/39 (5.1)	0.037; 4.89 (1.09–21.82)	0.302; 2.50 (0.43–14.38)
**Frequency of drinking alcohol**				
Rare/never	40/52 (76.9)	12/52 (23.1)	1.00	
Once a week	64/76 (84.2)	12/76 (15.8)	0.302; 1.60 (0.65–3.09)	0.076; 2.76 (0.90–8.51)
Diary	20/21 (95.2)	01/21 (4.8)	0.096; 6.00 (0.72–49.46)	0.109; 6.14 (0.66–56.83)
**Syphilis status** ^**b**^				
Negative	56/74 (75.7)	18/74 (24.3)	1.00	
Positive	68/75 (90.7)	07/75 (9.3)	0.018; 3.12 (1.21–8.00)	0.421; 1.63 (0.49–5.36)

NS: Not Significant; OR: Odds Ratio; 95% CI: 95% Confidence Interval

^#^National minimum wage: during the study period, one minimum wage represented approximately R$ 998.00 BRL (U$ 264.00 USD).

* Positivity for total anti-HAV antibodies

^**a**^Adjusted for age, education and monthly household income.

^**b**^ Serologic test for anti-*T*. *pallidum* antibodies by ELISA.

### HAV molecular analysis

**Fig 1:**
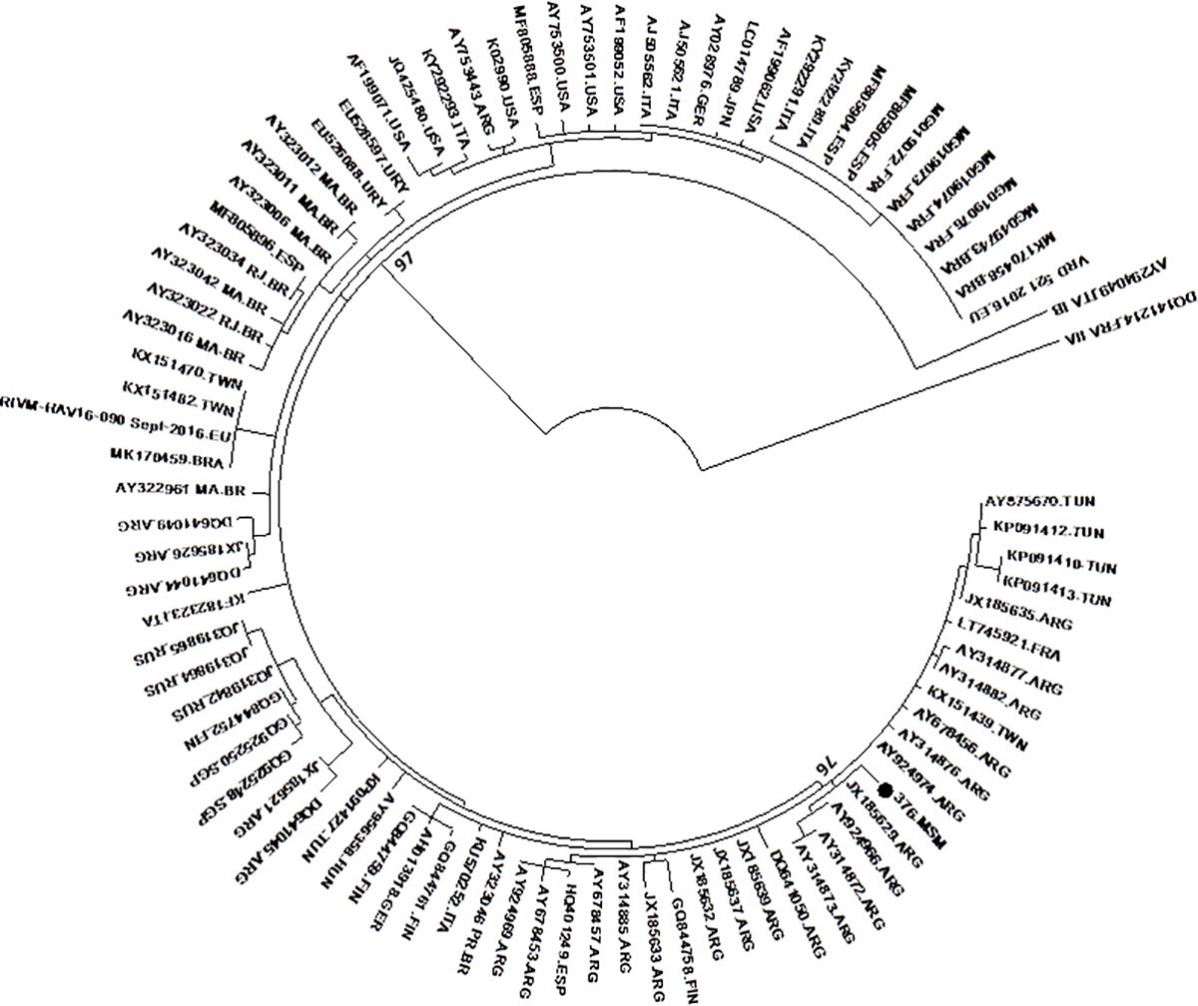
Maximum likelihood phylogenetic tree based on 85 HAV VP1-2A genotype IA sequences. In addition, sequences of genotypes IB and IIA were included as an outgroup. HAV sequence from this study.

The subject with evidence of present HAV infection (anti-HAV IgM positive) was a transgender woman, 20 years old, with active syphilis infection, irregular condom use and lived in a bordering region with Bolivia. Molecular analysis of HAV sequence revealed a close similarity (>99%) with subgenotype IA strains from Argentina and no relation to MSM European outbreak strains (Fig 1).

## Discussion

Efforts to control HAV MSM outbreaks worldwide have been focused on awareness-raising of risky sexual practices, reinforcing hygiene preventive measures, and in epidemiological surveillance on HAV dispersal patterns [5]. Sparse data on HAV prevalence in MSM and TW is available in Brazil, especially on the period before the men’s HAV outbreak (2017–2019). The present study is the first HAV investigation in a sample of MSM and TW in Central Brazil. The overall prevalence of anti-HAV (69.7%; 95% CI: 65.3–74.0) was high when compared to the general population of South and Southeast (33.7%; 95% CI, 32.4%–35.1%) [3], to a group of MSM from Poland (16.6%) [11], the Netherlands (37%; 95% CI, 35–40%) [12] and Italy (42.8%) [8]. Similar rates were found in individuals of North and Central-West regions (68.8%; 95% CI, 64.8%–72.5%), the latter being the same region of the present study. Similar prevalence was also observed in Afro-Brazilian rural communities from Central Brazil (75.6%; 95% CI: 72.7–78.3%) [13]. Areas more developed in socio-economic terms, as developed countries and South and Southeast regions of Brazil, present lower HAV prevalence [1, 3]. However, due to the men’s HAV outbreak between 2017–2019 in Brazilian southeastern states, some of these regions that had low HAV prevalence as a reflection of 2014 vaccination, have presented an upward trend, showing levels of infection similar to the pre-vaccine period [14].

Socioeconomic variable such as low household income was associated with anti-HAV positivity among MSM and TW in the present study, previously reported by other authors [1, 3]. In countries with high and moderate water-access rates, socioeconomic indicators are better predictors of HAV prevalence, even more than sanitation and water access conditions. In addition, as demonstrated by our study, ethnicity may play a role in anti-HAV positivity. As has been demonstrated, being non-white is a common barrier to STIs screening, facilitating the dispersal of HIV, hepatitis and other infections in this population [15].

The mean age among anti-HAV seropositive individuals was higher than in the seronegative group. Statistical analysis confirmed that increasing age was strongly associated with past HAV infection. This finding is consistent with previous studies and could be explained by environmental improvements, such as sanitary and socio-economic conditions that decrease the risk of infection along with the new generations [1, 8].

Sexual transmission is primarily responsible for the HAV spread among MSM and TW, according to previous studies [2, 4, 11, 12, 16, 17]. Despite no significant association that has been established with HAV exposure, high levels of risky sexual behaviors were found among MSM of the study, such as being a sex worker, multiple sexual partners, irregular condom use, and engagement in a variety of sexual practices. The sexual practice of rimming, defined by direct oro-anal contact, facilitates the spread of enteric pathogens among MSM [2, 9]. Fisting, shared use of sex toys and the presence of others STI as syphilis might lead to an increased risk of HAV transmission [8, 11]. Among MSM group, history of sexual violence showed a tendency to increase the anti-HAV prevalence in the multiple logistic regression analysis. As described previously, childhood sexual abuse was more experienced among MSM than heterosexual individuals and it may influence future risk behaviors [16].

Molecular analyses were performed in the transgender woman sample with serological evidence of present HAV infection. Subgenotype IA was detected, which is in accordance studies with MSM population [2]. However, phylogenetic analyses did not reveal a molecular link with the recent HAV outbreaks among MSM in some regions of Europe and Americas [4]. This sample was also not genetically related to other HAV strains circulating in Brazil, thus presenting high homology with a strain sampled in Rio de la Plata river, Argentina, in 2010 [18]. It is known the carrier is a sex professional, irregular condom user with active syphilis, who lived in a city bordering Bolivia. Thus, it is possible that this viral strain was acquired through unprotected sexual practices with an immigrant or traveler from Latin America. The high mobility, characteristic of sex workers, can support this hypothesis [19].

This study has some limitations. Firstly, the convenience-sampling method may have recruitment bias, restringing the representativeness of the entire MSM and TW communities. Secondly, other factors potentially relevant to HAV transmission were not addressed in this study, such as housing, sanitation, migration, and trips for endemic areas. Thirdly, although the molecular sequence identified here was similar to Argentine strains, more sequences would be necessary to achieve a better understanding of HAV molecular epidemiology in MSM and TW of Central Brazil. In addition, sampling selection was carried out a few years ago, thus the study may not reflect the current scenario of these group population. Despite these limitations, our study provided epidemiological data on HAV exposure and risk factors in key populations as MSM and TW. The recent HAV outbreaks in MSM and the increase in HAV prevalence in Brazil due to men’s outbreak demonstrate the need for continuous epidemiological surveillance. In addition, the study data revealed that in the study period there was no HAV outbreak in Central Brazil.

In conclusion, an intermediate prevalence of HAV exposure was found in this study, despite low socio-economic conditions. Although sexual transmission presents an important role in HAV spread in MSM and TW groups, unsafe sexual behaviors were not associated with HAV infection. The HAV sequence found in the study was similar to Argentine strains and not related to MSM European outbreak strains, as occurred in Chile and Southeast Brazil [4, 14, 20]. The declining trend in HAV incidence and the transition of the infection age may cause a major impact on the burden of the disease. To prevent outbreaks, such as one that occurred in São Paulo [6], vaccination programs targeting these populations could be considered as an important strategy to prevent this infection among MSM and TW in Central Brazil.

## References

[1] Jacobsen KH. Globalization and the Changing Epidemiology of Hepatitis A Virus. Cold Spring Harb Perspect Med. 2018 Oct 1;8(10):a031716. doi: 10.1101/cshperspect.a031716; PMCID: PMC6169986. 29500305 PMC6169986

[2] Vaughan G, Goncalves Rossi LM, Forbi JC, de Paula VS, Purdy MA, Xia G, et al. Hepatitis A virus: host interactions, molecular epidemiology and evolution. Infect Genet Evol. 2014 Jan;21:227–43. doi: 10.1016/j.meegid.2013.10.023 Epub 2013 Nov 5. . 24200587

[3] Ximenes RA, Martelli CM, Amaku M, Sartori AM, de Soárez PC, Novaes HM, et al. Modelling the force of infection for hepatitis A in an urban population-based survey: a comparison of transmission patterns in Brazilian macro-regions. PLoS One. 2014 May 20;9(5):e94622. doi: 10.1371/journal.pone.0094622; PMCID: PMC4028178. 24845598 PMC4028178

[4] World Health Organization (WHO). Hepatitis A outbreaks mostly affecting men who have sex with men–European Region and the Americas. Geneva: WHO; 2017. Available from: http://www.who.int/csr/don/07-june-2017-hepatitis-a/en/

[5] Williamson DA, Chen MY. Emerging and Reemerging Sexually Transmitted Infections. N Engl J Med. 2020 May 21;382(21):2023–2032. doi: 10.1056/NEJMra1907194 . 32433838

[6] Souto FJD, de Brito WI, Fontes CJF. Impact of the single-dose universal mass vaccination strategy against hepatitis A in Brazil. Vaccine. 2019 Feb 4;37(6):771–775. doi: 10.1016/j.vaccine.2018.12.054 Epub 2019 Jan 11. . 30639462

[7] Kerr L, Kendall C, Guimarães MDC, Salani Mota R, Veras MA, Dourado I, et al. HIV prevalence among men who have sex with men in Brazil: results of the 2nd national survey using respondent-driven sampling. Medicine (Baltimore). 2018 May;97(1S Suppl 1):S9–S15. doi: 10.1097/MD.0000000000010573 ; PMCID: PMC5991534. 29794604 PMC5991534

[8] Greco L, Uceda Renteria SC, Guarneri D, Orlandi A, Zoccoli A, Benardon S, et al. HEV and HAV seroprevalence in men that have sex with men (MSM): An update from Milan, Italy. J Med Virol. 2018 Aug;90(8):1323–1327. doi: 10.1002/jmv.25052 Epub 2018 May 10. . 29446470

[9] de Paula VS, Villar LM, Coimbra Gaspar AM. Comparison of four extraction methods to detect hepatitis A virus RNA in serum and stool samples. Braz J Infect Dis. 2003 Apr;7(2):135–41. doi: 10.1590/s1413-86702003000200007 . 12959685

[10] Hosmer D. W. Jr., Lemeshow S. & Sturdivant R. X. *Applied Logistic Regression*. (John Wiley & Sons, Inc., 2013).

[11] Polański P, Kucharczyk B, Kondej B, Cielebąk E, Kucharczyk A, Sadkowska-Todys M. Hepatitis A in Poland in 2017—epidemic increase cases. Przegl Epidemiol. 2019;73(4):487–497. doi: 10.32394/pe.73.46 . 32237698

[12] Alberts CJ, Boyd A, Bruisten SM, Heijman T, Hogewoning A, Rooijen MV et al. Hepatitis A incidence, seroprevalence, and vaccination decision among MSM in Amsterdam, the Netherlands. Vaccine. 2019 May 9;37(21):2849–2856. doi: 10.1016/j.vaccine.2019.03.048 Epub 2019 Apr 13. . 30992222

[13] Kozlowski Aline G et al. Prevalence of hepatitis A virus infection in Afro-Brazilian isolated communities in Central Brazil. Memórias do Instituto Oswaldo Cruz [online]. 2007, v. 102, n. 1 [Accessed 28 May 2021] doi: 10.1590/s0074-02762007000100021 17294012

[14] De Oliveira TM, Vieira NSG, Sepp TDS, Souto FJD. Recent trends in hepatitis A incidence in Brazil. J Med Virol. 2020 Aug;92(8):1343–1349. doi: 10.1002/jmv.25694 Epub 2020 Feb 10. . 32009240

[15] Siconolfi DE, Kapadia F, Halkitis PN, Moeller RW, Storholm ED, Barton SC, et al. Sexual health screening among racially/ethnically diverse young gay, bisexual, and other men who have sex with men. J Adolesc Health. 2013 May;52(5):620–6. doi: 10.1016/j.jadohealth.2012.10.002 Epub 2012 Dec 1. ; PMCID: PMC3634893. 23298989 PMC3634893

[16] Mayer KH, Bekker LG, Stall R, Grulich AE, Colfax G, Lama JR. Comprehensive clinical care for men who have sex with men: an integrated approach. Lancet. 2012 Jul 28;380(9839):378–87. doi: 10.1016/S0140-6736(12)60835-6 Epub 2012 Jul 20. ; PMCID: PMC5603076. 22819653 PMC5603076

[17] Mangla N, Mamun R, Weisberg IS. Viral hepatitis screening in transgender patients undergoing gender identity hormonal therapy. Eur J Gastroenterol Hepatol. 2017 Nov;29(11):1215–1218. doi: 10.1097/MEG.0000000000000950 . 28857896

[18] Blanco Fernández MD, Torres C, Riviello-López G, Poma HR, Rajal VB, Nates S, et al. Analysis of the circulation of hepatitis A virus in Argentina since vaccine introduction. Clin Microbiol Infect. 2012 Dec;18(12):E548–51. doi: 10.1111/1469-0691.12034 Epub 2012 Oct 17. . 23072283

[19] Bogart LM, Revenson TA, Whitfield KE, France CR. Introduction to the special section on Lesbian, Gay, Bisexual, and Transgender (LGBT) health disparities: where we are and where we’re going. Ann Behav Med. 2014 Feb;47(1):1–4. doi: 10.1007/s12160-013-9574-7 . 24327136

[20] Mello VM, Lago BV, Sousa PSF, Mello FCA, Souza CB, Pinto LCM, et al. Hepatitis A Strain Linked to the European Outbreaks During Gay Events between 2016 and 2017, Identified in a Brazilian Homosexual Couple in 2017. Viruses. 2019 Mar 20;11(3):281. doi: 10.3390/v11030281; PMCID: PMC6466027. 30897727 PMC6466027

